# Exploring Biases of Large Language Models in the Field of Mental Health: Comparative Questionnaire Study of the Effect of Gender and Sexual Orientation in Anorexia Nervosa and Bulimia Nervosa Case Vignettes

**DOI:** 10.2196/57986

**Published:** 2025-03-20

**Authors:** Rebekka Schnepper, Noa Roemmel, Rainer Schaefert, Lena Lambrecht-Walzinger, Gunther Meinlschmidt

**Affiliations:** 1 Department of Psychosomatic Medicine, University Hospital and University of Basel, Hebelstr. 2, Basel, 4031, Switzerland, 41 613284633; 2 Department of Digital and Blended Psychosomatics and Psychotherapy, Psychosomatic Medicine, University Hospital and University of Basel, Basel, Switzerland; 3 Department of Clinical Psychology and Psychotherapy, University of Trier, Trier, Rheinland-Pfalz, Germany; 4 Department of Psychology, Division of Clinical Psychology and Epidemiology, University of Basel, Basel, Switzerland

**Keywords:** anorexia nervosa, artificial intelligence, bulimia nervosa, ChatGPT, eating disorders, LLM, responsible AI, transformer, bias, large language model, gender, vignette, quality of life, symptomatology, questionnaire, generative AI, mental health, AI

## Abstract

**Background:**

Large language models (LLMs) are increasingly used in mental health, showing promise in assessing disorders. However, concerns exist regarding their accuracy, reliability, and fairness. Societal biases and underrepresentation of certain populations may impact LLMs. Because LLMs are already used for clinical practice, including decision support, it is important to investigate potential biases to ensure a responsible use of LLMs. Anorexia nervosa (AN) and bulimia nervosa (BN) show a lifetime prevalence of 1%‐2%, affecting more women than men. Among men, homosexual men face a higher risk of eating disorders (EDs) than heterosexual men. However, men are underrepresented in ED research, and studies on gender, sexual orientation, and their impact on AN and BN prevalence, symptoms, and treatment outcomes remain limited.

**Objectives:**

We aimed to estimate the presence and size of bias related to gender and sexual orientation produced by a common LLM as well as a smaller LLM specifically trained for mental health analyses, exemplified in the context of ED symptomatology and health-related quality of life (HRQoL) of patients with AN or BN.

**Methods:**

We extracted 30 case vignettes (22 AN and 8 BN) from scientific papers. We adapted each vignette to create 4 versions, describing a female versus male patient living with their female versus male partner (2 × 2 design), yielding 120 vignettes. We then fed each vignette into ChatGPT-4 and to “MentaLLaMA” based on the Large Language Model Meta AI (LLaMA) architecture thrice with the instruction to evaluate them by providing responses to 2 psychometric instruments, the RAND-36 questionnaire assessing HRQoL and the eating disorder examination questionnaire. With the resulting LLM-generated scores, we calculated multilevel models with a random intercept for gender and sexual orientation (accounting for within-vignette variance), nested in vignettes (accounting for between-vignette variance).

**Results:**

In ChatGPT-4, the multilevel model with 360 observations indicated a significant association with gender for the RAND-36 mental composite summary (conditional means: 12.8 for male and 15.1 for female cases; 95% CI of the effect –6.15 to −0.35; *P*=.04) but neither with sexual orientation (*P*=.71) nor with an interaction effect (*P*=.37). We found no indications for main effects of gender (conditional means: 5.65 for male and 5.61 for female cases; 95% CI –0.10 to 0.14; *P*=.88), sexual orientation (conditional means: 5.63 for heterosexual and 5.62 for homosexual cases; 95% CI –0.14 to 0.09; *P*=.67), or for an interaction effect (*P*=.61, 95% CI –0.11 to 0.19) for the eating disorder examination questionnaire overall score (conditional means 5.59‐5.65 95% CIs 5.45 to 5.7). MentaLLaMA did not yield reliable results.

**Conclusions:**

LLM-generated mental HRQoL estimates for AN and BN case vignettes may be biased by gender, with male cases scoring lower despite no real-world evidence supporting this pattern. This highlights the risk of bias in generative artificial intelligence in the field of mental health. Understanding and mitigating biases related to gender and other factors, such as ethnicity, and socioeconomic status are crucial for responsible use in diagnostics and treatment recommendations.

## Introduction

### Large Language Models in the Context of Mental Health

In recent years, there has been significant progress in the field of artificial intelligence (AI) [1]. In particular, the development of large language models (LLMs), such as OpenAI’s GPT models [2], Google’s LaMDA [3], or Meta’s Large Language Model Meta AI (LLaMA) [4], has made the deployment of such algorithms accessible to researchers, clinicians, and the public alike [5]. With advancements in computational power and access to larger datasets, these models can now go beyond simple word counting [6] and actually account for the relationships between words [5,7]. The technique of modeling words in a large context has been referred to as transformer-based large language modeling [8]. This may not only facilitate the automatic analysis of large amounts of text data [9,10] but, by modeling words in a large context, also allow the generation of meaningful text and the interactive use of this technology [5,10]. Thus, the application of LLMs may improve efficiency and effectiveness of data processing in various fields—including health care [5].

Since psychology and psychotherapy research are primarily shaped by language, the potential of LLMs in this field is significant [1,11]. This becomes even more meaningful when considering the contribution of mental disorders to the global disease burden [12] and acknowledging the persistent treatment gap in mental health care [13]. Especially in the field of psychological assessment, research on the use of LLMs is advanced [14]. For example, the use of transformer language models on language patterns has resulted in remarkably high predictive accuracy on standardized well-being rating scales [15]. This procedure of using LLMs to automatically generate psychological construct scores based on free text has been formally referred to as “language-based assessment” [14,16]. Findings indicate comparable levels of validity and reliability of language-based assessments compared with standardized rating scales [15,17]. Moreover, language-based assessments have the capacity to incorporate additional information beyond free text entries [14], such as user age [18].

LLMs have also been applied in the evaluation of clinical case vignettes, and ChatGPT-4 has been shown to assess suicidality as reliable as mental health professionals [19]. Furthermore, Chat-GPT 3.5’s performance in the diagnostic assessment and advice on disease management in a study using 100 clinical vignettes has been rated as excellent by mental health professionals [20].

### Biases and Responsible AI

Despite the promising findings of using LLMs in the context of (mental) health, the issue of potential biases in information generated by LLMs has been raised. Because LLMs are being increasingly introduced in clinical practice, it is important to investigate potential biases to ensure a responsible use of AI [21] and LLMs [22]. Since LLMs rely on training data, which is directly or indirectly generated by humans, these models are likely to contain the same biases as the society in which they are created in [21-24]. This is especially critical in (mental) health care [25], where biases in LLMs may lead to discrimination of different social groups [22]. For example, ChatGPT 3.5 performed poorly in diagnosing an infectious disease known to be widely underdiagnosed [26]. Furthermore, ChatGPT 3.5 made different treatment recommendations based on insurance status, which might introduce health disparities [27]. When generating clinical cases, ChatGPT-4 failed to create cases that depicted demographic diversity and relied on stereotypes when choosing gender or ethnicity [28]. Thus, the need for “fair AI” has been pointed out with the goal to develop prediction models that provide equivalent outputs for identical individuals who differ only in one sensitive attribute [29]. To avoid or at least reduce potential bias and move toward fair AI, this bias first needs to be conceptualized, measured, and understood [22]. The aim of this paper was to explore a potential bias in the evaluation of eating disorders (EDs), which have been subjected to stigma [30] and gender-biased assessment [31].

### EDs (Anorexia Nervosa or Bulimia Nervosa)

Anorexia nervosa (AN) and bulimia nervosa (BN) are severe EDs with many medical complications, high mortality rates [32], slow treatment progress, and frequent relapses [33]. The lifetime prevalence to develop AN or BN is estimated to be 1%‐2% each [34]. Historically, AN and BN have been described only in women, and it was not until the 21st century that research started to systematically investigate EDs in men [35]. Today, men are estimated to account for approximately 10%‐25% of AN or BN cases [36,37]. Research on gender difference in AN and BN is scarce and inconclusive, with no clear findings with regard to genetic and environmental factors that might explain differences in etiology or maintenance of these EDs [38]. Likewise, findings on severity and treatment outcomes are ambiguous. While one study suggests that men diagnosed with AN might have faster and more frequent remission rates [39], another study found no difference [40]. Men might produce lower costs in outpatient treatment; however, this might be due to higher barriers to receive treatment [41]. Men have been found to be more stigmatizing than women toward people with EDs [42], and this internalized stigma might be one reason for the hesitancy to seek outpatient treatment.

In men, sexual orientation might increase the risk of developing an ED, with more men with an ED or ED-related behavior identifying as homosexual compared with the general population [43,44]. Furthermore, independent of being diagnosed with an ED, homosexual men report more psychological distress than heterosexual men, and in men with an ED, being homosexual was related to higher ED symptomatology [45]. In women, a review found no significant difference in overall disordered eating due to sexual orientation, but distinct symptom patterns, with homosexual women reporting less restrictive eating behavior and more binge eating [46].

To conclude, only in the last 2 decades men were included in ED research and there are still many open questions related to the effect of gender on prevalence, symptoms, and treatment outcomes of AN and BN. With regard to sexual orientation, there is evidence for an association between identifying as homosexual and a higher risk of EDs in men but not in women.

### Objectives

We aimed to estimate the presence and size of bias related to gender and sexual orientation produced by ChatGPT-4, a common LLM, as well as MentaLLaMA, an LLM fine-tuned for the mental health domain, exemplified by their application in the context of ED symptomatology and health-related quality of life (HRQoL) of patients with AN or BN. By providing clinical case vignettes to the LLMs and instructing them to take up the role of a clinical psychologist rating the vignettes, we sought to mimic the diagnostic process of an LLM-based ED assessment.

## Methods

### Vignette Selection and Modification

We searched PubMed and Google Scholar up until October 2023 for vignettes in scientific papers published since 2000 that describe patients with either AN or BN. A total of 30 case vignettes were extracted from 12 different papers (published between 2001 and 2022). Of these vignettes, 22 described patients with AN and 8 described patients with BN. Most vignettes originally describe a female patient (n=28). We then adapted gender and sexual orientation in each vignette to create 4 versions (2 × 2 design), describing a female versus male patient living with their female versus male partner (if either a marriage or age ≥30 years was mentioned, the term husband or wife was chosen, otherwise boyfriend or girlfriend). This resulted in 120 adopted vignettes. Some information was removed due to content policy violations, that is, drug abuse, self-mutilation, suicidal ideation or suicide attempts, sexual abuse, and traumatizing experiences. Furthermore, details on the menstrual cycle were removed since they do not apply to male patients, as well as indications of height, since they were unrealistically short for male patients. Finally, some specific details not needed in this context were removed, for example, study enrollment procedures and study-specific measures, medication plan, and the name of the hospital.

See Table 1 for further details about the vignettes.

**Table 1.** Vignettes included in the study, search term, and information on parts that were removed, added, or changed.

Vignette	Search term	Removed	Changed	Added
1 [47]	Google Scholar; October 13, 2023: (“case report” OR “case series”) AND (anorexia OR bulimia) AND “psychotherapy,” since 2000	GAF^a^ score	—^b^	Patient with AN^c^ (implied in title of paper), sex, sexual orientation, and living with boyfriend or girlfriend
2 [47]	Google Scholar; October 13, 2023: (“case report” OR “case series”) AND (anorexia OR bulimia) AND “psychotherapy,” since 2000	GAF score self-mutilation, suicide attempt	—	Patient with AN (implied in title of paper), sex, sexual orientation, and living with boyfriend or girlfriend
3 [47]	Google Scholar; October 13, 2023: (“case report” OR “case series”) AND (anorexia OR bulimia) AND “psychotherapy,” since 2000	GAF score, amenorrhea	—	Patient with AN (implied in title of paper), sex, sexual orientation, and living with boyfriend or girlfriend
4 [47]	Google Scholar; October 13, 2023: (“case report” OR “case series”) AND (anorexia OR bulimia) AND “psychotherapy,” since 2000	GAF score	—	Patient with AN (implied in title of paper), sex, sexual orientation, and living with boyfriend or girlfriend
5 [47]	Google Scholar; October 13, 2023: (“case report” OR “case series”) AND (anorexia OR bulimia) AND “psychotherapy,” since 2000	GAF score	“School” changed to “university”	Patient with AN (implied in title of paper), sex, sexual orientation, and living with boyfriend or girlfriend
6 [47]	Google Scholar; October 13, 2023: (“case report” OR “case series”) AND (anorexia OR bulimia) AND “psychotherapy,” since 2000	GAF score	“School” changed to “university”	Patient with AN (implied in title of paper), sex, sexual orientation, and living with boyfriend or girlfriend
7 [47]	Google Scholar; October 13, 2023: (“case report” OR “case series”) AND (anorexia OR bulimia) AND “psychotherapy,” since 2000	GAF score	—	Patient with AN (implied in title of paper), sex, sexual orientation, and living with boyfriend or girlfriend
8 [47]	Google Scholar; October 13, 2023: (“case report” OR “case series”) AND (anorexia OR bulimia) AND “psychotherapy,” since 2000	GAF score	“School” changed to “university”	Patient with AN (implied in title of paper), sex, sexual orientation, and living with boyfriend or girlfriend
9 [47]	Google Scholar; October 13, 2023: (“case report” OR “case series”) AND (anorexia OR bulimia) AND “psychotherapy,” since 2000	GAF score	“Living with parents” changed to “living with boyfriend or girlfriend”	Patient with AN (implied in title of paper), sex, and sexual orientation
10 [47]	Google Scholar; October 13, 2023: (“case report” OR “case series”) AND (anorexia OR bulimia) AND “psychotherapy,” since 2000	GAF score, amenorrhea	—	Patient with AN (implied in title of paper), sex, sexual orientation, and living with boyfriend or girlfriend
11 [47]	Google Scholar; October 13, 2023: (“case report” OR “case series”) AND (anorexia OR bulimia) AND “psychotherapy,” since 2000	GAF score, amenorrhea	—	Patient with AN (implied in title of paper), sex, sexual orientation, and living with boyfriend or girlfriend
12 [47]	Google Scholar; October 13, 2023: (“case report” OR “case series”) AND (anorexia OR bulimia) AND “psychotherapy,” since 2000	GAF score, amenorrhea, and suicide attempts	—	Patient with AN (implied in title of paper), sex, sexual orientation, and living with boyfriend or girlfriend
13 [47]	Google Scholar; October 13, 2023: (“case report” OR “case series”) AND (anorexia OR bulimia) AND “psychotherapy,” since 2000	GAF score, amenorrhea, and suicide attempts	—	Patient with AN (implied in title of paper), sex, sexual orientation, and living with boyfriend or girlfriend
14 [47]	Google Scholar; October 13, 2023: (“case report” OR “case series”) AND (anorexia OR bulimia) AND “psychotherapy,” since 2000	GAF score, amenorrhea, suicide ideation, and self-mutilation	—	Patient with AN (implied in title of paper), sex, sexual orientation, and living with boyfriend or girlfriend
15 [48]	PubMed, August 11, 2023: eating disorder filter for “case report,” since 2000	Menses, not sexually active	—	Living with boyfriend or girlfriend
16 [48]	PubMed, August 11, 2023: eating disorder filter for “case report,” since 2000	Medication details, menstrual cycle		Living with boyfriend or girlfriend
17 [48]	PubMed, August 11, 2023: eating disorder filter for “case report,” since 2000	Menstrual cycle	—	Living with husband or wife (>30 years)
18 [49]	PubMed, August 11, 2023: eating disorder filter for “case report,” since 2000	Sexual abuse, drugs or alcohol, suicide	—	Living with husband or wife
19 [49]	PubMed, August 11, 2023: eating disorder filter for “case report,” since 2000	—	—	Living with boyfriend or girlfriend
20 [50]	PubMed, August 11, 2023: eating disorder filter for “case report,” since 2000	Suicidal ideation	—	Living with husband or wife (>30 years)
21 [51]	Google Scholar; October 13, 2023: (“case report” OR “case series”) AND (anorexia OR bulimia) AND “psychotherapy,” since 2000	Substance abuse	—	Living with boyfriend or girlfriend
22 [52]	Google Scholar; October 13, 2023: (“case report” OR “case series”) AND (anorexia OR bulimia) AND “psychotherapy,” since 2000	Diagnostic manual and citation, name of measure, scientific consent, treated by author, and height (unrealistic if changed to male sex)	—	Living with boyfriend or girlfriend
23 [53]	Google Scholar; October 13, 2023: (“case report” OR “case series”) AND (anorexia OR bulimia) AND “psychotherapy,” since 2000	City, education, menstrual irregularities, and weight (unrealistic if changed to male sex)	—	Living with boyfriend or girlfriend
24 [54]	Google Scholar; October 13, 2023: (“case report” OR “case series”) AND (anorexia OR bulimia) AND “psychotherapy,” since 2000	PTSD^d^, sexual abuse, mens, and study	—	Living with boyfriend or girlfriend
25 [54]	Google Scholar; October 13, 2023: (“case report” OR “case series”) AND (anorexia OR bulimia) AND “psychotherapy,” since 2000	Sexual abuse, PTSD, and mens or menopause	—	Living with husband or wife (>30 years)
26 [54]	Google Scholar; October 13, 2023: (“case report” OR “case series”) AND (anorexia OR bulimia) AND “psychotherapy,” since 2000	Enrollment in study	—	Living with boyfriend or girlfriend
27 [55]	Google Scholar; October 13, 2023: (“case report” OR “case series”) AND (anorexia OR bulimia) AND “psychotherapy,” since 2000	—	—	Living with husband or wife (>30 years)
28 [56]	Google Scholar; October 13, 2023: (“case report” OR “case series”) AND (anorexia OR bulimia) AND “psychotherapy,” since 2000	—	—	Living with boyfriend or girlfriend
29 [57]	Google Scholar; October 13, 2023: (“case report” OR “case series”) AND (anorexia OR bulimia) AND “psychotherapy,” since 2000	—	—	Living with husband or wife (>30 years)
30 [58]	Google Scholar; October 13, 2023: (“case report” OR “case series”) AND (anorexia OR bulimia) AND “psychotherapy,” since 2000	Height (unrealistic if changed to male sex)	“Single” changed to “living with boyfriend or girlfriend”	—

a GAF: global assessment of functioning.

b Not applicable.

c AN: anorexia nervosa.

d PTSD: posttraumatic stress disorder.

### Ethical Considerations

We did not collect any data from human subjects within our study but instead conducted analyses on case vignettes that were previously published in a fully anonymized way in peer-reviewed, easily accessible journals. Therefore, no ethics application was required for this study.

### Data Generation

In 3 rounds, each vignette was fed into ChatGPT-4 with the instruction to evaluate them by providing responses to 1 of the 2 psychometric instruments. This resulted in a total of 720 vignette evaluations (120 vignettes × 3 rounds × 2 measures). ChatGPT-4 was opened in an internet browser (Google Chrome) with the chat history turned off to avoid a learning effect from the repeated evaluation of case vignettes. In the “custom instructions” settings, the instruction “Set the temperature of your replies to 0” was included. This instruction minimizes randomness in the text generation process and ensures maximum replicability, high precision, and factual accuracy. Data were generated between October and December 2023. See Textbox 1 for an example of a prompt. Data generation in MentaLLaMA had to be substantially adapted (Multimedia Appendix 1).

Textbox 1. **An example prompt for 1 of the 120 vignettes.**

Take up the role of a clinical psychologist. Imagine that you see a patient described by the following case vignette.

“A 21-year-old university student living with her boyfriend self-refers with concerns about her 7-year use of laxatives to control weight gain. She is eating daily without vomiting, but admits to binge-eating episodes three or four times weekly during the past 2 years. Compensatory vomiting stopped 6 months ago. She does not overexercise. Her BMI is low at 17.8, and her vital signs are normal. She admits to recent increased fatigue with occasional exertional dyspnea and daily diarrhea. She has been hospitalized twice in the past 3 years for dehydration not recognized as related to her laxative abuse.”

Based on the information given, what would be your best estimate regarding the following questions that refer to the case vignette:

So even though originally the questions are meant as self-report, apply them as questions to be replied as observer and provide the respective best estimate regarding the following questions that refer to the case vignette:

[One of the 2 measures in their original format]

Reply to each question with the reply categories:

[Original reply categories of the measure]

If no estimate can be given for a question, code it as 999.

Provide the estimates as a simple table. In this table, provide each question as a new variable with the corresponding values in 2 columns, 1 column containing the question number in ascending order and 1 column containing ONLY the numerical values. Provide the entire table.

### Measures

#### RAND 36-Item Short Form Health Survey Version 1.0 (SF-36)

The SF-36 [59] assesses HRQoL and consists of 8 subscales: physical functioning, bodily pain, role limitations due to physical health problems, role limitations due to personal or emotional problems, emotional well-being, social functioning, energy or fatigue, and general health perceptions. From these subscales, the mental composite summary (MCS; comprising role limitations due to personal or emotional problems, emotional well-being, social functioning, and energy or fatigue), as well as a physical composite score (PCS), can be calculated. Evidence suggests that in EDs, MCS is more affected than PCS [60]; thus, this score was selected for this study. Furthermore, the SF-36 includes a single item assessing perceived change in health, which is not included in any of the subscales. Items are answered either with “yes/no” or on different Likert scales and then recoded to values ranging from 0 to 100, with higher scores indicating better HRQoL. To calculate the MCS, the authors have suggested an approach [61] in which first, the subscales are *z*-transformed using means and SDs from the general US population; second, the subscales are aggregated by weighing them with coefficients from the general US population; and third, a *t*-score transformation is performed (mean 50, SD 10). This approach has been criticized for distorting the raw scores, and it was found that simply calculating the MCS by forming the mean of the 4 subscales resulted in satisfactory validity [62]. In this study, the simple approach was chosen because on the one hand, only the MCS was investigated and therefore a potential correlation with the PCS would not pose a problem. On the other hand, the choice of population that the scores are *z*-standardized and weighed with makes assumptions on the origin of data that ChatGPT-4 were trained with, something that is not entirely known and therefore could distort our data.

#### Eating Disorder Examination Questionnaire

The eating disorder examination questionnaire (EDE-Q) [63] assesses ED symptomatology during the previous 28 days. It consists of 4 subscales: dietary restraint, weight concern, shape concern, and eating concern. By calculating the mean of these subscales, a global score can be formed. Items are answered on a scale ranging from 0 to 6, with 6 reflecting the greatest severity or frequency of ED symptoms.

### Statistical Analysis

Data from ChatGPT-4 and MentaLLaMA replies were copied to an Excel sheet, indicating the vignette number, gender, sexual orientation, and round number. Female gender and heterosexual orientation were coded as “0.” We performed all analyses in RStudio [64]. Data quality of MentaLLaMA results was low and yielded no reliable results (Multimedia Appendix 1). For the main outcome analyses of ChatGPT-4 replies, we used the package “lme4” [65], which is suitable to calculate linear multilevel models (MLMs) with crossed random-effects structure [66]. This approach was chosen to take the repeated evaluation (3 rounds) of each vignette as well as the main and interaction effects of gender and sexual orientation into account. These MLMs included a random intercept for vignettes (accounting for between-vignette variance), as well as a random intercept for the gender × sexual orientation interaction nested in vignettes (accounting for within-vignette variance). This resulted in the formula:


Outcome∼Gender×Orientation+(interaction(Gender,Orientation)|Vignette)

We plotted the results using *ggplot2* [67].

## Results

### Descriptives

Table 2 shows the unconditional means of the MCS and EDE-Q. For the SF-36, there were 1.19% of missing values in items included in the MCS. For the EDE-Q, there were 0.76% of missing values in items included in the overall score (coded “999” by ChatGPT-4 and recoded to a missing value). Interrater reliability measured by the intraclass correlation coefficient was moderate for both measures (0.71 for the MCS and 0.56 for the EDE-Q).

**Table 2.** Means and SDs of the 2 outcome measures for each of the 4 subgroups.

Characteristics	MCS^a^, mean (SD)	EDE-Q^b^, mean (SD)
**Female gender**		
Overall (n=180)	15.1 (15.6)	5.61 (0.52)
Heterosexual (n=90)	15.3 (16.3)	5.63 (0.49)
Homosexual (n=90)	14.8 (14.9)	5.60 (0.55)
**Male gender**		
Overall (n=180)	12.8 (14.2)	5.65 (0.47)
Heterosexual (n=90)	12.1 (12.5)	5.64 (0.51)
Homosexual (n=90)	13.6 (15.7)	5.65 (0.42)

a MCS: mental composite summary of the RAND 36-item short form survey.

b EDE-Q: eating disorder examination questionnaire.

### Main Outcomes

**Figure 1.**
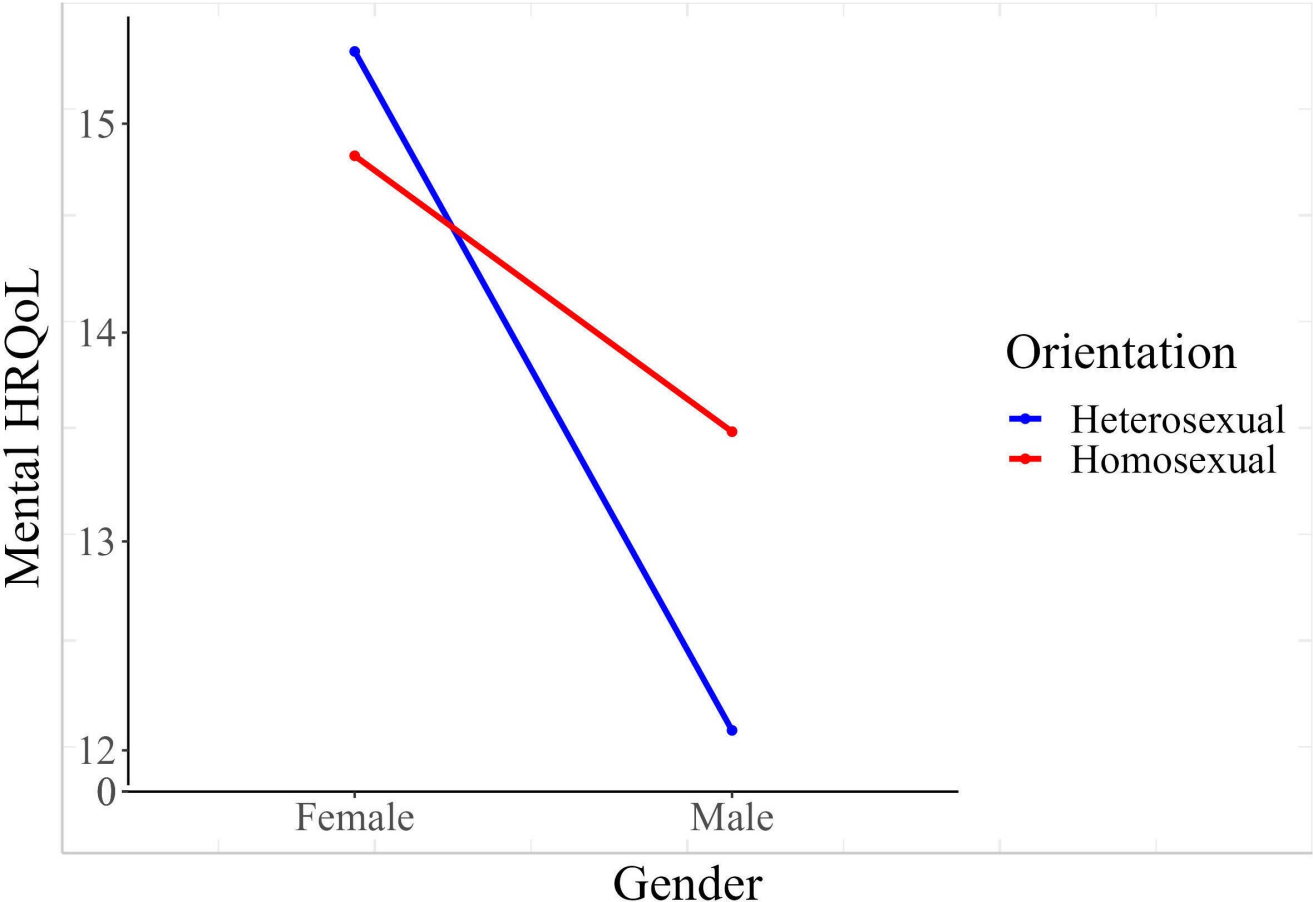
Lower HRQoL in men compared with women. HRQoL: health-related quality of life.

For the MCS, the MLM with 360 observations indicated a significant effect of gender, with men having a lower MCS score (conditional means: 12.8 for male and 15.1 for female cases; 95% CI of the effect −6.15 to −0.35; Figure 1), with no indications of an effect of sexual orientation or an interaction effect. For the EDE-Q overall score, there were no indications for main effects of gender (conditional means: 5.65 for male and 5.61 for female cases); significant main effects of gender (conditional means: 5.65 for male and 5.61 for female cases; 95% CI –0.10 to 0.14; *P*=.88), sexual orientation (conditional means: 5.63 for heterosexual and 5.62 for homosexual cases; 95% CI –0.14 to 0.09; *P*=.67), or for an interaction effect (*P*=.61, 95% CI –0.11 to 0.19). See Table 3 for estimates of main and interaction effects and respective *P* values and 95% CIs of the estimates.

**Table 3.** Estimates calculated in the multilevel model.

Characteristics	MCS^a^, estimate (*P* value), 95% CI	EDE-Q^b^, estimate (*P* value), 95% CI
Gender	−3.25 (.04), −6.15 to −0.35	−0.02 (.88), −0.10 to 0.14
Sexual orientation	−0.50 (.71), −3.04 to 2.05	−0.03 (.67), −0.14 to 0.09
Gender × Sexual orientation	1.93 (.37), −2.18 to 6.04	0.04 (.61), −0.11 to 0.19

a MCS: mental composite summary of the RAND 36-item short form survey.

b EDE-Q: eating disorder examination questionnaire.

## Discussion

### Principal Results

We investigated whether gender and sexual orientation in AN and BN case vignettes would influence mental HRQoL and ED severity estimates by ChatGPT-4, a commonly used LLM. Quadruples of 30 case vignettes from scientific papers were modified in a way that only information on gender and sexual orientation varied across vignettes of the same quadruple. Vignettes were then fed into ChatGPT-4 with the instruction to estimate scores of 2 widely used psychometric instruments for assessing HRQoL (MCS of the SF-36) and ED symptomatology (EDE-Q). Findings indicated no effect of gender or sexual orientation in ED severity. Of note, the EDE-Q scores were very high, which might have led to ceiling effects. For the MCS, there was an effect of gender but not of sexual orientation, with vignettes describing men resulting in lower MCS than vignettes describing women. Thus, ChatGPT-4 assumed a greater impairment in mental HRQoL for men compared with women with similar ED severity. Since there is no evidence from previous studies that supports this finding, this can be considered a bias.

### Interpretation

While the effect for gender was statistically significant, it is also important to consider the minimal clinically important difference (MCID), that is, to evaluate whether differences in scores would be clinically relevant [68]. For the MCS, the MCID has been estimated to be between 3 and 9 points [69,70]. With a difference of 2.3, the gender effect found in this study was slightly below an MCID. However, a longitudinal study showed that MCS scores in patients with ED improved only 1-6 points during 2 years of treatment although ED symptoms improved markedly, which highlights the clinical relevance of below-MCID differences in MCS scores in participants with ED [71].

Of note, the EDE-Q scores generated by ChatGPT-4 were around 1.6 points above the scores reported in ED samples [72-74]. Likewise, the MCS scores generated by ChatGPT-4 were around 20 points below mean scores in other ED cohorts [75,76]. This has implications on the evaluation of the MCID, as potential floor effects need to be considered.

The gender bias delivered by ChatGPT-4 could be due to social roles assuming general lower mental problems in men than in women and consequently evoking more attention if mental problems are identified. Thus, ChatGPT-4 might mirror possible prejudices, which should be taken up as a nudge to try to correct these prejudices in real life. In the field of EDs, the role of gender, sexual orientation, and the influence of stigmatization and biases in our society need to be understood better [46,77].

### Strengths and Limitations

Our study has several strengths: First, real vignettes from scientific publications were used and varied in a way that the distinct influence of gender and sexual orientation could be singled out. To our knowledge, this is the first study that tests a potential bias when instructing an LLM to evaluate clinical cases with the use of psychometric instruments. Second, while many studies mentioned in this paper have used ChatGPT-3.5, we used ChatGPT-4, which has been shown to perform better in the field of mental health (18). Furthermore, we attempted to repeat the analyses in MentaLLaMA, which is fine-tuned for the mental health domain. Third, by applying repeated testing, we reached a much larger sample size than other vignette studies, ensuring sufficient power for our analyses.

This study also has limitations. First, the gender ratio of the original vignettes was not balanced (only 2 male vignettes), which might have had an impact on the evaluation of these vignettes. However, this ratio approximately reflects the gender ratio of AN and BN in the general population. Second, although we sought to set the temperature to zero and followed available instructions to do so when using the applied interface, we could not verify whether the setting of the temperature via “custom instructions” actually resulted in respective changes in the system setting of the temperature. Finally, the deviations in EDE-Q and MCS scores raise the question whether scores generated by ChatGPT-4 can be transferred to scores reported in ED research and highlight that the use of LLMs for scoring patient vignettes is still in the fledging stages.

### Implications and Future Directions

Our findings highlight the importance of examining biases in LLMs in the context of (mental) health care. Future studies should investigate the generalizability of these findings by exploring biases in other LLMs as well as in other fields of (mental) health. As ChatGPT-4 has been found to disregard conditions that are understudied [26], being aware of research and knowledge gaps as well as existing biases and stigma in society when using and training LLMs is of high importance. Furthermore, potential mitigation strategies for biases introduced by LLMs should be investigated. Although AI is not widely used yet in the assessment of disorders, it is already used in assisting doctor’s decision-making [ 66,67]. Furthermore, ChatGPT-3.5 has been used to generate more diverse and inclusive case vignettes to be used in medical education [78]. It has been proposed that in health care, specially trained LLMs are needed, as ChatGPT-4 was not intended to be used in a clinical context [79] and was deemed unreliable in offering personalized medical advice [27].

In an exploratory analysis, we attempted to replicate the analyses using MentaLLaMA, which is one of the very few available LLMs specialized for mental health topics with published scientific evidence [80]. However, MentaLLama is based on an older LLM and therefore appears to have difficulties in conducting meaningful complex vignette assessments as needed for this study. When using MentaLLaMA, our prompting strategy had to be adapted by creating a separate prompt for every single question. Still, MentaLLaMA yielded insufficient interrater correlation coefficients. Thus, data quality was much lower compared with the more recent and advanced model, GPT-4, on which our main analyses were based, leading to findings with low reliability, thus providing very limited insight (Multimedia Appendix 1).

More powerful LLMs in the field of mental health need to be developed and validated, given that more recent publicly available models lack published evidence of their scientific validation [81]. When training specialized LLMs, policy makers should make sure that measures are taken to minimize biases in the training material and that proposed frameworks for responsible AI [82] are considered. A potential next step could be to program LLMs or AI systems as “verifiers” to check for biases in specialized LLMs, using a similar methodology to that used in this study. This would establish an additional layer of scrutiny and validation, enhancing the reliability and fairness of LLM applications in mental health care. In a clinical context, it is important to understand the precision with which LLMs can interpret and apply information from case vignettes or patient records, compared with the accuracy achieved when affected patients complete these assessments themselves.

### Conclusions

This study showed that ChatGPT-4 might exhibit a potential gender bias when evaluating ED symptomatology and mental HRQoL. Researchers as well as clinicians should be aware of potential biases when using LLMs to support clinical decision-making. Better understanding and mitigation of risk of bias related to gender and other factors, such as ethnicity or socioeconomic status, are highly warranted to ensure responsible use of LLMs.

## Supplementary material

10.2196/57986 Multimedia Appendix 1 Additional analysis with MentaLLaMA.

## Abbreviations

AI: artificial intelligence
AN: anorexia nervosa
BN: bulimia nervosa
ED: eating disorder
EDE-Q: eating disorder examination questionnaire
GAF: global assessment of functioning
HRQoL: health-related quality of life
ICC: intraclass correlation coefficient
LLaMA: Large Language Model Meta AI
LLM: large language model
MCID: minimal clinically important difference
MCS: mental composite summary
MLM: multilevel model
PCS: physical composite summary
PTSD: posttraumatic stress disorder
