# Supplementary material for: Exploring Biases of Large Language Models in the Field of Mental Health: Comparative Questionnaire Study of the Effect of Gender and Sexual Orientation in Anorexia Nervosa and Bulimia Nervosa Case Vignettes
Source: JMIR Ment Health. 2025 Mar 20;12:e57986. doi: 10.2196/57986 (PMC11949086; doi:10.2196/57986)
Supplement: Multimedia Appendix 1 [file mental-v12-e57986-s001.docx]

**Supplement 1**

As an additional analysis, we ran a mental health-specific large language models on a local machine. We utilized the model “MentaLLaMA)” [1], based on the Meta LLaMA2-chat-7B foundation model, retrieved via the model platforms of “Ollama” (https://ollama.com/vitorcalvi/mentallama2). MentaLLaMA is based on the Large Language Model Meta AI (LLaMA) architecture [2], with 6.7 billion parameters and a context length of 4,096 tokens. We used quantized versions of the models, using Q4_0 schemes. We selected MentaLLaMA based on evidence that it is one of the best performing available LLMs out of all LLMs that i) are finetuned specifically on mental health domain data, and ii) have been assessed and are supported by published evidence [e.g., 3].

Our computational environment consisted of a MacBook Pro 14" (2023) with an Apple M2 Max processor, 64 GB of RAM, running macOS 13.3.1. We conducted the analyses using Python 3.11.5 and Ollama 0.3.11. We developed a Python script to automate the process of reading clinical vignettes, questions, and answer options, using them in a series of prompts – one separate prompt for each vignette-version-question combination – as input to the LLM and returning separate LLM output to each prompt, to subsequently be parsed and stored. The script interfaced with the LLMs via Ollama, sending the formatted prompts and receiving the models’ outputs. We had to adapt our prompt that we originally used with the GPT-4 model to generated usable output with the above-mentioned LLM: We designed the prompt to instruct the model to “Act as if you were a clinical psychologist” and presented each of the questions required to calculate the respective score, together with the vignette version, along with predefined answer options for the models to choose from to the LLM. We included explicit instructions for the LLM to provide their answer by selecting one of the given options and to use an exact format: “Answer Number/Option: [number/option]” followed by “Answer Text: [text]” (‘number’ mentioned for SF-36 and ‘option’ mentioned for EDE-Q items, as the reply option of the letter are marked with alphabetic characters). To ensure consistency and facilitate parsing of the responses, we emphasized that the LLM should not include any reasoning, explanations, or additional text beyond the specified format. Additionally, we accounted for potential mismatches between the vignette and the question by instructing the LLM to make the best possible guess without commenting on any discrepancies. We implemented a parsing function to extract the answer numbers/options and texts from the models’ responses, ensuring they matched the expected format. In case that the model did not provide a valid response in form of at least an answer number/option, no result was exported. Valid results were exported to an Excel file for subsequent analyses.

Means and standard deviations are shown in supplementary table 1. Notably, inter-rater reliability measured by the intraclass correlation coefficient (ICC) was poor for both measures (0.37 for the EDE-Q and 0 for the MCS). ANOVAs revealed no significant group differences for the MCS (all *p*s>.199) or the EDE-Q (all *p*s>.448). Due to these unambiguously non-significant values, no further multilevel models (MLMs) were calculated.

Table S1. Means and standard deviations of the two outcome measures for each of the four subgroups using “MentaLLaMA”.

|  | MCS^a^, mean score (SD) | EDE-Q^b^, mean score (SD^c^) |
| --- | --- | --- |
|  |  |  |
| **Female Gender** |  |  |
| Overall (*n*=180) | 33.0 (5.74) | 4.22 (0.24) |
| Heterosexual (*n*=90) | 33.4 (5.07) | 4.21 (0.23) |
| Homosexual (*n*=90) | 32.6 (6.33) | 4.23 (0.25) |
| **Male Gender** |  |  |
| Overall (*n*=180) | 32.9 (6.10) | 4.23 (0.27) |
| Heterosexual (*n*=90) | 33.3 (6.34) | 4.22 (0.30) |
| Homosexual (*n*=90) | 32.5 (5.86) | 4.24 (0.25) |

^a^ Mental composite summary of the RAND 36-item short form survey

^b^ Eating disorder examination questionnaire

^c^ Standard deviation

**References**

1. Yang, K., et al., *MentaLLaMA: Interpretable Mental Health Analysis on Social Media with Large Language Models*, in *Proceedings of the ACM Web Conference 2024*. 2024, Association for Computing Machinery: Singapore, Singapore. p. 4489–4500.

2. Touvron, H., et al., *Llama: Open and efficient foundation language models.* arXiv preprint arXiv:2302.13971, 2023.

3. Adhikary, P.K., et al., *Exploring the Efficacy of Large Language Models in Summarizing Mental Health Counseling Sessions: Benchmark Study.* JMIR Ment Health, 2024. **11**: p. e57306.
